# Branching into brains

**DOI:** 10.7554/eLife.33066

**Published:** 2017-12-05

**Authors:** Adam Shai, Matthew Evan Larkum

**Affiliations:** 1 Department of BiologyStanford UniversityStanfordUnited States; 2 Neurocure Cluster of ExcellenceHumboldt UniversityBerlinGermany

**Keywords:** deep learning, dendritic morphology, neocortex, credit assignment, feedback alignment, target propagation, None

## Abstract

What can artificial intelligence learn from neuroscience, and vice versa?

**Related research article** Guerguiev J, Lillicrap TP, Richards BA. 2017. Towards deep learning with segregated dendrites. *eLife* **6**:e22901. doi: 10.7554/eLife.22901

Deep learning is a subfield of machine learning that focuses on training artificial systems to find useful representations of inputs. Recent advances in deep learning have propelled the once arcane field of artificial neural networks into mainstream technology (LeCun et al., 2015). Deep neural networks now regularly outperform humans on difficult problems like face recognition and games such as Go (He et al., 2015; Silver et al., 2017). Traditional neuroscientists have also taken an interest in deep learning because it seemed initially that there were telling analogies between deep networks and the human brain. Nevertheless, there is a growing impression that the field might be approaching a new ‘wall’ and that deep networks and the brain are intrinsically different.

Chief among these differences is the widely held belief that backpropagation, the learning algorithm at the heart of modern artificial neural networks, is biologically implausible. This issue is so central to current thinking about the relationship between artificial and real brains that it has its own name: the credit assignment problem. The error in the output of a neural network (that is, the difference between the output and the 'correct' answer) can be reported or 'backpropagated' to any connection in the network, no matter where it is, to teach the network how to refine the output. But for a biological brain, neurons only receive information from the neurons they are connected to, making credit assignment a real problem. How does the brain blindly adjust the strength of the connections between neurons that are far removed from the output of the network? In the absence of a solution, we may be forced to conclude that deep learning and brains are incompatible after all.

Now, in eLife, Jordan Guerguiev, Timothy Lillicrap and Blake Richards propose a biologically inspired solution to the credit assignment problem (Guerguiev et al., 2017). Central to their model is the structure of the pyramidal neuron, which is the most prevalent cell type in the cortex (the outer layer of the brain). Pyramidal neurons have been a source of aesthetic pleasure and interesting research questions for neuroscientists for decades. Each neuron is shaped like a tree with a trunk reaching up and dividing into branches near the surface of the brain as if extending toward a source of energy or information. Can it be that, while most cells of the body have relatively simple shapes, evolution has seen to it that cortical neurons are so intricately shaped as to be apparently impractical?

Guerguiev et al. – who are based at the University of Toronto, the Canadian Institute for Advanced Research, and DeepMind – report that this impractical shape has an advantage: the long branched structure means that error signals at one end of the neuron and sensory input at the other end are kept separate from each other. These sources of information can then be brought together at the right moment in order to find the best solution to a problem.

As Guerguiev et al. note, many facts about real neurons and the structure of the cortex turn out to be just right to find optimal solutions to problems. For instance, the bottoms of cortical neurons are located just where they need to be to receive signals about sensory input, while the tops of these neurons are well placed to receive feedback error signals (Cauller, 1995; Larkum, 2013). The key to this design principle seems to be to keep these distinct information streams largely independent. At the same time, ion channels under the control of a host of other nearby neurons process and gate the transfer of information within the neuron.

Taking inspiration from these facts Guerguiev et al. implement a deep network with units that have different compartments, just like real neurons, that can separate sensory input from feedback error signals. These units have all the information they need to know in order to nudge the network toward the desired output. Guerguiev et al. prove formally that this approach is mathematically sound. Moreover, their new, biologically plausible deep network is able to perform well on a task to identify handwritten numbers, and does so by creating what are referred to as hierarchical representations. This phenomenon refers to the increasingly complex nature of the responses of the network's layers, commonly found in more traditional deep learning models, and in the sensory cortices of biological brains.

Doubtless, there will be more twists and turns to this story as more biological details are incorporated into the model. For instance the brain also faces a time-based credit assignment problem (Friedrich et al., 2011; Gütig, 2016). Guerguiev et al. admit that this network does not outperform non-biologically derived deep networks – yet. Nevertheless, the model they present paves the way for future work that links biological networks to machine learning. The hope is that this can be a two-way process, in which insights from the brain can be used to improve artificial intelligence, and insights from artificial intelligence can be used to reveal how the brain operates.
